# Injury factors and pathological features of toxic milk mice during different disease stages

**DOI:** 10.1002/brb3.1459

**Published:** 2019-11-19

**Authors:** Xiang‐xue Zhou, Xun‐hua Li, Ding‐bang Chen, Chao Wu, Li Feng, Hao‐lin Qin, Xiao‐Yong Pu, Xiu‐ling Liang

**Affiliations:** ^1^ Department of Neurology The East Area of the First Affiliated Hospital Sun Yat‐Sen University Guangzhou China; ^2^ Department of Neurology The First Affiliated Hospital Sun Yat‐Sen University Guangzhou China; ^3^ Department of Radiology The East Area of the First Affiliated Hospital Sun Yat‐Sen University Guangzhou China; ^4^ Department of Reproductive Medicine and Urology Guangdong General Hospital Guangdong Academy of Medical Sciences Guangzhou China

**Keywords:** injury factors, pathological characteristics, susceptibility‐weighted images, toxic milk mice, Wilson's disease

## Abstract

**Objective:**

To evaluate different injury factors and pathological characteristics of the brain at different disease stages in toxic milk (TX) mice, an animal model of Wilson's disease (WD).

**Methods:**

Thirty TX mice (10 each at 3, 6 and 12 months old) and 30 age‐matched C57 mice were used in this study. Corrected phase (CP) values were determined from susceptibility‐weighted images. Myelin content was determined by measuring inhibition optical density values of Luxol fast blue‐stained sections. Neurofilament protein 68 kDa (NF68), β‐amyloid precursor protein (β‐APP), and myelin basic protein (MBP) levels, as well as copper and iron content, in brain nuclei of the TX mouse were evaluated. Gene amplification ratios for catalase (CAT), GSH peroxidase (GSH‐PX), nitric oxide synthase (NOS), and superoxide dismutase (SOD) in mouse brain were also determined.

**Results:**

Compared with C57 mice, neuronal cell counts were decreased in 12‐months‐old TX mice (*p* = .011). Myelin content was decreased in the lenticular nucleus (*p* = .029), thalamus (*p* = .030), and brainstem (*p* = .034) of 6‐months‐old TX mice; decreases in the corresponding nuclei (*p* = .044, .037, and .032, respectively) were also found in 12‐months‐old TX mice. MBP values were lower in the lenticular nucleus and thalamus (*p* = .027 and .016, respectively) of 6‐months‐old TX mice and in the corresponding nuclei (*p* = .24 and .040) of 12‐months‐old TX mice. NF‐68 values were lower in the lenticular nucleus and thalamus (*p* = .034 and .037, respectively) of 6‐months‐old TX mice and in the corresponding nuclei (*p* = .006 and .012) of 12‐months‐old TX mice. β‐APP values were higher in the thalamus of 6‐months‐old (*p* = .037) and 12‐months‐old (*p* = .012) TX mice. Iron content was higher in the lenticular nucleus, thalamus, and cerebellum (*p* = .044, .038, and .029, respectively) of 6‐months‐old TX mice and in the corresponding nuclei (*p* = .017, .024, and .029) of 12‐months‐old TX mice. The NOS gene amplification multiple was higher (*p* = .039), whereas the SOD1 gene amplification multiple was lower (*p* = .041) in 12‐months‐old TX mice. There was no correlation between metal content or oxidation index and pathological index.

**Conclusions:**

The pathological characteristics of the brains of TX mice may differ at different ages. Different pathogenic factors, including copper and iron deposition and abnormal oxidative stress, are present at different stages.

## Graphical abstract

The brain pathological characteristics of toxic milk (TX) mice at different age of months may be different. Both copper and iron deposition, abnormal oxidative stress may be pathogenic factors.

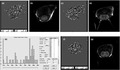

## LIMITATIONS

This study has a number of limitations. First, because ROIs were drawn by radiologists relative to anatomical structures, there may have been random variability in the measurements. To reduce this variability, two neuroradiologists independently made all measurements, with the reported value for each being an average of three different measurements. Second, copper and iron content were evaluated in homogenates of tissue cut to the same volume; thus, these values do not fully represent metal deposition in the whole nuclei. To reduce this variability, we averaged results from three tissue samples to obtain a value of metal content for each nucleus.

## 1 INTRODUCTION

Wilson's disease (WD) is an autosomal recessive inherited disorder of copper metabolism characterized by excessive copper deposition, especially in liver and brain. Toxic milk (TX) mice, an animal model of WD harboring an A4066G point mutation in ATP7B (ATPase copper transporting beta), can be used for researching the pathology of WD.

Regardless of the disease stage of WD patients, there is only one clinical option for treatment of WD: copper excretion therapy. Moreover, there is no consensus on whether there are different pathological changes in the brain at different stages of WD disease or other causes besides copper deposition. If there are different pathological changes and/or pathogenic factors in the brain at different stages of WD, different treatment plans should be adopted according to the specific pathological changes and pathogenic factors. Accordingly, the purpose of this study was to identify pathological damage characteristic of different disease stages and analyze the pathogenic factors associated with different stages in the brains of TX mice, an animal model of WD.

Copper deposition has long been considered the only pathogenic cause of WD. Ceruloplasmin, a copper‐containing plasma ferroxidase, plays an essential role in iron metabolism (Hellman & Gitlin, 2002). Low ceruloplasmin levels and reduced ferroxidase activity may lead to storage of iron in WD. Recent studies have shown that WD may be complicated by high iron concentrations in basal ganglia of the brain (Kim et al., 2005; Sorbello, Sini, Civolani, & Demelia, 2010). However, iron deposition in WD remains to be established. In addition to metal deposition, it is not clear whether other pathogenic factors, such as oxidative stress injury, are present in WD. Whether these risk factors differ in different stages of disease has also not been studied. It is similarly unknown whether characteristic pathological changes in WD, which include demyelination, axonal injury, and neuronal necrosis, are different in different stages of the disease.

Susceptibility‐weighted imaging (SWI) is a sensitive method for detecting metals, particularly iron, that has been used for in vivo assessment of brain metal concentrations (Rossi, Ruottinen, Soimakallio, Elovaara, & Dastidar, 2013). Abnormal SWI signals in the brains of WD patients have been previously reported (Lee et al., 2012; Zhou et al., 2014). However, the characteristics of metal deposition in WD on SWI and the utility of this technique for evaluating copper deposition require further investigation. Importantly, whether SWI results reflect pathologic features of WD is not known. Currently, pathological processes can be investigated using a variety of quantitative methods. Demyelination is analyzed histologically using Luxol fast blue (LFB) myelin staining (Sheng‐Kwei Song et al., 2005), and molecularly by measuring levels of myelin basic protein (MBP), a component of myelin (Gonzalez‐Gronowa et al., 2015). Axonal damage is quantified by measuring axonal swelling by immunostaining for β‐amyloid precursor protein (β‐APP; Sheng‐Kwei Song et al., 2005) and neurofilament protein 68 kDa (NF68; Lapin et al., 2015; Posmantur, Newcomb, Kampfl, & Hayes, 2000).

In the current study, we sought to evaluate the different injury factors and pathological characteristics in different nuclei of the brains of TX mice at different disease stages using pathological and SWI methods.

## 2 MATERIALS AND METHODS

### 2.1 Animals

Thirty TX mice (10 each at 3, 6 and 12 months old) and 30 age‐matched C57 mice were used in this study. Mice were maintained in the animal housing facility of the East Area of the First Affiliated Hospital, Sun Yat‐Sen University. All procedures were performed in accordance with the guidelines of the Institutional Animal Care and Use Committee of the university.

### 2.2 Molecular resonance imaging (MRI) protocol

Anesthesia was induced via intraperitoneal injection of barbiturate (100 mg/kg). All animals underwent SWI tests of the brain. MRI data were obtained using an Achieva Nova Dual 3.0 T scanner (Philips Healthcare) equipped with eight‐channel phased‐array coils. A human finger coil was used for acquisition of imaging data. SWI images were obtained parallel to the anteroposterior commissural line using a high‐resolution, gradient‐echo sequence with the following parameters: TR/TE, 60/40 ms; flip angle, 18°; slices, 48; field of view, 230/184 mm; matrix, 240 × 240. All data were transferred to a workstation (Philips extended MR workspace 2.6.3.2; Philips Medical Systems) for offline analysis. Regions of interest (ROIs) included the lenticular nucleus, thalamus, brain stem, and cerebellum, and were placed by two radiologists according to the rat atlas. Values of the corrected phase (CP) of ROIs were verified on SWI. Random variability in measurements was reduced by computing each value as an average of three different measurements.

### 2.3 Histological studies

Histological analyses were performed on all mice. After euthanizing animals with an overdose of barbiturate, brains were quickly removed and fixed with paraformaldehyde or frozen. Tissues containing the lenticular nucleus, thalamus, brain stem, and cerebellum were sliced into 2‐µm‐thick sections. Nerve cells were Nissl‐stained and quantified by averaging measurements obtained in 3–5 fields per slide. Myelin was evaluated in paraffin‐embedded sections stained with Luxol fast blue (LFB) and quantified by assessing inhibition optical density (IOD) values of LFB‐stained sections, where high IOD values indicate high myelin content. MBP levels were evaluated using enzyme‐linked immunosorbent assays (ELISAs). Frozen sections were immunostained separately for NF68 and β‐APP by incubating first with chicken anti‐NF68 and rabbit anti‐β‐APP antibodies, respectively, and then with goat anti‐chicken or anti‐rabbit polyclonal secondary antibody. The number of axons was estimated in both NF68 and β‐APP‐stained slides, and IOD values of sections were determined.

NF68 and β‐APP were quantified by Western blotting. Briefly, proteins were separated by sodium dodecyl sulfate‐polyacrylamide gel electrophoresis (SDS‐PAGE) at 100 V for 1 hr and transferred to polyvinylidene fluoride membranes. All membranes were incubated with chicken anti‐NF68 or rabbit anti‐β‐APP primary antibody, followed by incubation at room temperature for 1 hr with horseradish peroxidase (HRP)‐conjugated anti‐chicken or anti‐rabbit secondary antibody. Immunoreactive proteins were detected using enhanced chemiluminescence (ECL) with subsequent exposure of membranes to radiographic film. NF68 and β‐APP proteins levels were quantified by scanning optical densitometry. Gene amplification ratios for catalase (CAT), GSH peroxidase (GSH‐PX), nitric oxide synthase (NOS), and superoxide dismutase (SOD) in the mouse brain were determined by reverse transcription‐polymerase chain reaction. Copper and iron content of nuclei were determined by flame atomic absorption spectrometry of tissue homogenates (tissue volume, 0.5 × 0.5 × 0.5 cm).

### 2.4 Statistical analysis

Results are presented as means ± *SEM*. Statistical analyses were performed using SPSS 13.0. (SPSS Inc.). Parameters of TX and C57 mice were compared using independent sample *t* tests (two‐tailed *t* test). Associations between histological parameters and metal content were assessed by Pearson's correlation analysis. *p*‐values <.05 were considered statistically significant.

## 3 RESULTS

### 3.1 CP values

**Figure 1:**
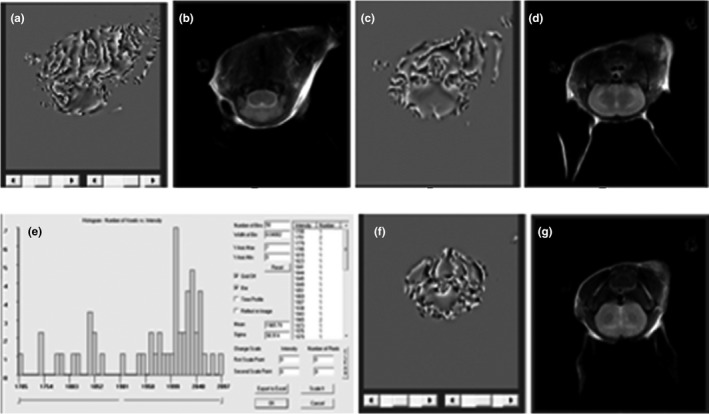
Image processing of SWI in TX mice. (a) SWI image of brain stem in TX mice; (b) MRT2 image of brain stem in TX mice; (c) SWI image of thalamic in TX mice; (d) MRT2 image of thalamus of TX mice; (e) SWI CP analysis chart; (f) SWI image of lentiform nucleus in TX mice; (g) MRT2 image of lentiform nucleus in TX mice

Corrected phase values were significantly different between 3‐months‐old TX and control mice in the lentiform nucleus (*p* = .023). At 6 months of age, CP values for TX mice were lower than those for control mice in the lentiform nucleus (*p* = .033) and thalamus (*p* = .021). CP values for 12‐months‐old TX mice were lower than those of control mice in the lentiform nucleus (*p* = .041), thalamus (*p* = .029), brain stem (*p* = .043), and cerebellum (*p* = .039; Table 1, Figure 1).

**Table 1:** CP values in TX and C57 mice

ROI	Lentiform nucleus	Thalamus	Brain stem	Cerebellum	Cortex
TX mice					
3 months old	1,834 ± 74*	1,946 ± 23	1,918 ± 129	1,955 ± 11	2,165 ± 77
6 months old	1,777 ± 123*	1,824 ± 218*	1,887 ± 115	1,922 ± 93	2,174 ± 96
12 months old	1,723 ± 96*	1,807 ± 77*	1,833 ± 75*	1,907 ± 153*	2,039 ± 214
C57 mice					
3 months old	2,025 ± 119	2,022 ± 50	2,006 ± 98	2,110 ± 72	2,182 ± 13
6 months old	2,018 ± 70	2,015 ± 97	2,010 ± 69	2,092 ± 51	2,198 ± 34
12 months old	2,047 ± 29	2,005 ± 43	2,037 ± 112	2,133 ± 207	2,146 ± 55

Note

Data are presented as means ± standard deviation.

* Statistically significant compared to C57 mice (*p *≤ .05).

### 3.2 Pathology findings

**Figure 2:**
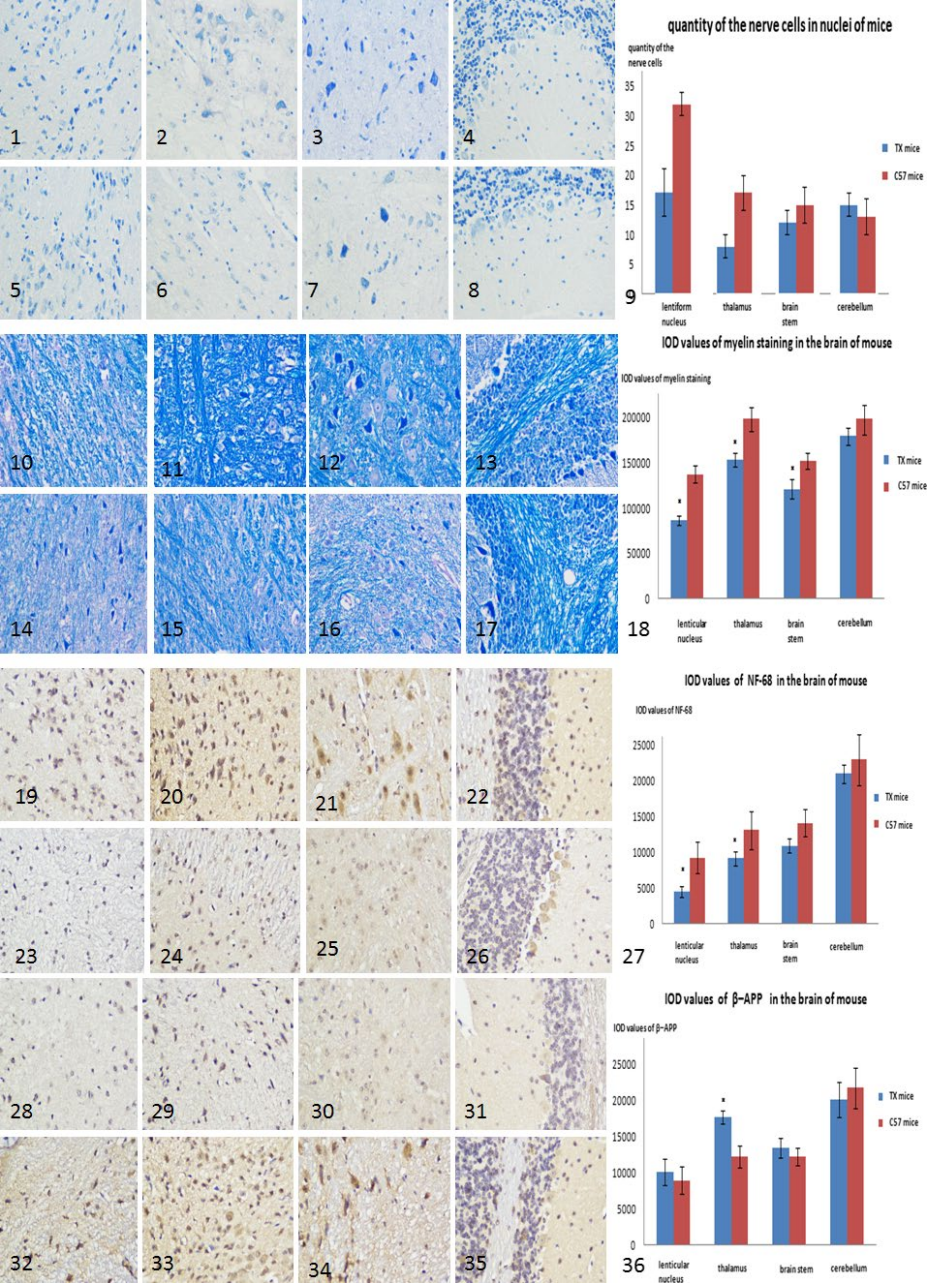
Nissl staining in the lenticular nucleus (1), thalamus (2), brain stem (3), cerebellum (4) of C57 mice. Nissl staining in the lenticular nucleus (5), thalamus (6), brain stem (7), cerebellum (8) in TX mice of 12 months old. The nerve cell counts in the lenticular nucleus and thalamus decreased in TX of 12 months old than C57 mice (9). Sections of C57 mice with LFB staining in the lenticular nucleus (10), thalamus (11), brain stem (12), cerebellum (13), and sections in TX mice of 6 months old with LFB staining in the lenticular nucleus (14), thalamus (15), brain stem (16), and cerebellum (17) are shown. IOD values of LFB staining were significantly lower in the lenticular nucleus (*p* = .029), thalamus (*p* = .030), and brainstem (*p* = .034) of TX than in C57 mice (18). Images of NF68 staining in the lenticular nucleus (19), thalamus (20), brain stem (21), and cerebellum (22) of C57 mice, and NF68 staining in the lenticular nucleus (23), thalamus (24), brain stem (25), and cerebellum (26) of TX mice of 6 months old are shown. IOD values of NF‐68 staining were significantly lower in the lenticular nucleus (*p* = .034) and thalamus (*p* = .037) in TX on 6 months old than in C57 mice (27). Images of β‐APP staining in the lenticular nucleus (28), thalamus (29), brain stem (30), and cerebellum (31) of C57 mice, β‐APP staining in the lenticular nucleus (32), thalamus (33), brain stem (34), and cerebellum (35) in TX mice of 6 months old are shown. IOD values of β‐APP staining were significantly higher in the thalamus (*p* = .037) in TX of 6 months old than in C57 mice (36). ※: *p* ≤ .05 compared with C57 mice

**Figure 3:**
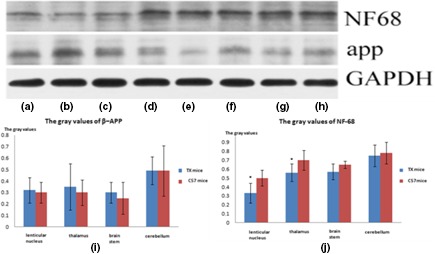
Western blot of β‐APP and NF‐68 in the lenticular nucleus (a), thalamus (b), brain stem (c), and cerebellum (d) in TX mice of 6 months old and lenticular nucleus (e), thalamus (f), brain stem (g), cerebellum (h) in C57 mice are shown. Gray values of WB of β‐APP (i). Gray values of NF‐68 were significantly lower in the lenticular nucleus (*p* = .035) and thalamus (*p* = .040) in TX of 6 months old than in C57 mice (j). ※: *p* ≤ .05 compared with C57 mice

No significant differences in nerve cell counts between TX and C57 mice were observed at 3 or 6 months of age. However, nerve cell counts were decreased in 12‐months‐old TX mice compared with C57 mice (*p* = .011). Myelin content, determined by measuring IOD values for LFB‐stained sections, was significantly lower in the lenticular nucleus (*p* = .029), thalamus (*p* = .030), and brainstem (*p* = .034) of 6‐months‐old TX mice compared with those in C57 mice; decreases in myelin content in the corresponding nuclei (*p* = .044, .037, and .032, respectively) were also found in 12‐months‐old TX mice (Figure 2). MBP values were lower in the lenticular nucleus and thalamus (*p* = .027 and .016, respectively) of 6‐months‐old TX mice and in the corresponding nuclei (*p* = .24 and .040) of 12‐months‐old TX mice compared with C57 mice (Table 2). IOD values for NF‐68 were lower in the lenticular nucleus and thalamus (*p* = .034 and .037, respectively) of 6‐months‐old TX mice and in the corresponding nuclei (*p* = .006 and .012) of 12‐months‐old TX mice compared with C57 mice (Figure 2). IOD values for β‐APP were higher in the thalamus of 6‐months‐old (*p* = .037) and 12‐months‐old (*p* = .012) TX mice compared with C57 mice (Figure 2). Gray values for NF‐68 were significantly lower in the lenticular nucleus and thalamus of TX mice at 6 months old (*p* = .035 and .040, respectively) and 12 months old (*p* = .016 and .029, respectively) compared with C57 mice (Figure 3).

**Table 2:** MBP values in brains of mice (pg/ml)

	Lentiform nucleus	Thalamus	Brain stem	Cerebellum	Cortex
TX mice of 6 months old	80 ± 11*	80 ± 10*	97 ± 19	114 ± 22	59 ± 9
TX mice of 12 months old	77 ± 25*	70 ± 21*	95 ± 32	110 ± 17	52 ± 13
C57 mice	107 ± 22	120 ± 11	105 ± 24	124 ± 12	66 ± 15

Note

Data are presented as means ± standard deviation.

* Statistically significant compared to healthy controls (*p *≤ .05).

Copper content was significantly higher in the lenticular nucleus (*p* = .031) and thalamus (*p* = .039) of 3‐months‐old TX mice; it was also significantly higher in the lenticular nucleus, thalamus, brain stem, cerebellum, and cortex (*p* = .027, .011, .040, .016, and .025, respectively) of 6‐months‐old TX mice and in the corresponding brain nuclei (*p* = .017, .022, .011, .041, .044) of 12‐months‐old TX mice compared with C57 mice (Table 3). Iron content was higher in the lenticular nucleus (*p* = .044), thalamus (*p* = .038), and cerebellum (*p* = .029) of 6‐months‐old TX mice, and was also significantly increased in the corresponding regions (*p* = .017, .024, .029) of 12‐months‐old TX mice compared with C57 mice (Table 3).

**Table 3:** Metal parameters in nuclei of mice

	Months	Lentiform nucleus		Thalamus		Brain stem		Cerebellum		Cortex	
		Copper	Iron	Copper	Iron	Copper	Iron	Copper	Iron	Copper	Iron
TX mice (μmol/L)	3	1.03 ± 0.12*	1.52 ± 0.42	1.00 ± 0.23*	1.60 ± 0.47	0.29 ± 013	2.72 ± 0.76	0.20 ± 0.10	1.76 ± 0.47	0.20 ± 0.14	1.27 ± 0.76
	6	1.05 ± 0.07*	2.60 ± 0.39*	1.06 ± 0.11*	2.70 ± 0.21*	1.03 ± 0.04*	2.90 ± 0.52	0.93 ± 0.10*	2.30 ± 0.32*	0.78 ± 0.13*	1.22 ± 0.21
	12	1.12 ± 0.23*	2.72 ± 0.27*	1.17 ± 0.11*	2.97 ± 0.55*	1.17 ± 0.33*	2.84 ± 0.76	0.87 ± 0.49*	2.42 ± 0.59*	0.88 ± 0.36*	1.20 ± 0.47
C57 mice (μmol/L)	3	0.26 ± 0.07	1.57 ± 0.03	0.30 ± 0.07	1.69 ± 0.21	0.25 ± 0.06	2.12 ± 0.22	0.18 ± 0.07	1.52 ± 0.37	0.18 ± 0.04	1.22 ± 0.26
	6	0.27 ± 0.04	1.56 ± 0.03	0.28 ± 0.06	1.60 ± 0.04	0.22 ± 0.02	2.06 ± 0.53	0.21 ± 0.03	1.50 ± 0.33	0.18 ± 0.06	1.18 ± 0.70
	12	0.27 ± 0.06	1.59 ± 0.06	0.29 ± 0.09	1.54 ± 0.19	0.39 ± 0.09	2.22 ± 0.47	0.22 ± 0.05	1.50 ± 0.26	0.19 ± 0.01	1.19 ± 0.23

Note

Data represent means ± standard deviation.

* Statistically significant compared to C57 mice (*p *≤ .05).

Nitric oxide synthase gene amplification multiples were higher (*p* = .039), whereas SOD1 gene amplification multiples were lower (*p* = .041) in 12‐months‐old TX mice compared with age‐matched C57 mice (Table 4).

**Table 4:** Influencing factors of CP values in TX mice

CP values	Factors	*B*	*p*
Lenticular nucleus	Copper content	−4.132	.012
	Iron content	−50.500	.010
Thalamus	Copper content	−2.107	.032
	Iron content	−13.200	.040
Brain stem	Iron content	−111.688	.013
Cerebellum	Iron content	−231.760	.041
Cortex	Copper content	−131.826	.039

### 3.3 Correlations between pathological or metal parameters and SWI parameters

There was no correlation between copper or iron content and IOD values for LFB staining, NF‐68, or β‐APP. Pathological indicators were not correlated with oxidative stress indicators.

Pathological parameters and CP values were not significantly correlated.

Negative correlations between CP values and iron (*r* = −.753, *p* = .032) and copper (*r* = −.260, *p* = .046) content were evident in TX mice.

## 4 DISCUSSION

Wilson's disease is an autosomal recessive defect of cellular copper export. Brain lesions of WD patients are usually symmetrical, involving the globus pallidus, putamen, claustrum, thalamus, cortical and subcortical regions, mesencephalon, pons, and cerebellum (Jadav et al., 2013; Prashanth et al., 2010). At present, only a single copper excretion treatment scheme is employed for different disease stages of WD. But the types of metal deposits in WD brain are not entirely clear. Copper accumulation in the brain is obvious, but is difficult to quantitatively evaluate. Additionally, the issue of whether iron deposition contributes to brain damage in WD remains to be established. Accordingly, it is important to find effective methods for evaluating metal deposition in WD. In addition to metal deposition, it is not clear whether WD brain injury is caused by other injury factors, such as oxidative stress. If other injury factors besides metal deposition can be found, it will significantly impact the search for new therapeutic targets. In addition, it is not clear whether pathological changes in the brain are different at different stages of WD. Clearly, understanding the pathological characteristics of different disease stages would be helpful in making stage‐specific treatment decision. Therefore, the purpose of our study was to study the pathological characteristics and different pathogenic factors of different disease stages in the brain of TX mice, an animal model of WD.

To quantify myelin content, we performed histological analyses using LFB‐PAS staining. MBP was additionally measured as means for detecting demyelination. We observed a decrease in LFB myelin staining and increased MBP content in subcortical nuclei of TX mice at age 6 and 12 months, indicating demyelination in these regions. Axonal degeneration was assessed by quantifying NF‐68 and β‐APP levels using immunohistochemistry and Western blotting. NF‐68 is an important structural component of neuronal axons (Posmantur et al., 2000), whereas β‐APP is transferred along axons. Axonal injury was detected in 6‐ and 12‐months‐old TX mice, as evidenced by a decrease in NF‐68 and an increase in β‐APP content. The number of nerve cells was also significantly decreased in 12‐months‐old TX mice, indicating nerve cell damage at this age. The above results suggest that brain pathological features characteristics of TX mice differ at different ages. At an early stage of the disease (3 months old), there may be no significant pathological damage. In the middle stage of disease (6 months old), demyelination and axonal injury were clearly evident in the brains of TX mice, but no obvious neuronal necrosis was found. In the late stage of disease (12 months old), obvious necrosis of nerve cells was detected in the TX mouse brain.

Abnormal iron deposition in the brain has been observed in many chronic diseases (Han et al., 2013; Zhang et al., 2009). Consistent with this, we found increases in both copper and iron content in subcortical nuclei of TX mice. Ceruloplasmin, a potent ferroxidase that catalyzes the conversion of ferrous to ferric iron, is essential for iron transport across the cell membrane (Zhang et al., 2009). A ceruloplasmin deficiency may result in brain iron overload. Iron deposition may also result in secondary neuronal degeneration in WD (Hingwala, Kesavadas, Thomas, & Kapilamoorthy, 2013; Rossi et al., 2013). In the early stages of the disease (3 months old), abnormal deposits of copper are present in subcortical nuclei. The deposition of copper and iron in the brains of TX mice increases gradually with increasing age. In addition, there are differences in metal deposition between different sites. Notably, metal deposition was significantly higher in subcortical nuclei than in the cortex.

In addition to metal deposition, other damage factors may be present in the WD brain. Excessive NO, generated by copper‐dependent up‐regulation of inducible NOS (iNOS; Kumar et al., 2012), can inhibit the mitochondrial respiratory chain and cause cell death (Phani, Loike, & Przedborski, 2012). The glutathione (GSH) system, which includes reduced GSH and GSH peroxidase (GSH‐PX), is an important free radical scavenging system in the brain (Valente, Arena, Torosantuccil, & Gelmetti, 2012). Superoxide dismutase (SOD) is one of the key enzymes involved in protecting cells from oxidative stress (Cury et al., 2016). The hydroxyl radical (OH‐), the most active oxygen species, can be decomposed by catalase (CAT). Therefore, CAT levels determine the degree of oxidative stress. We found that expression of the NOS gene in 12‐months‐old TX mice was higher than normal, suggesting an abnormally increased oxidative stress response. However, GSH‐PX in 12‐months‐old TX mice was lower than normal, indicating decreased antioxidant capacity. The above results suggest the presence of abnormal oxidative stress in TX mice at the late stage of disease (12 months old), indicating that this may be another pathogenic factor involved in WD nerve injury.

Hypointense signals in SWI may reflect mineral deposition (Lee et al., 2012). Although a negative correlation between changes in the SWI signal and iron accumulation has been confirmed (Zhang et al., 2009), the utility of SWI in evaluating copper deposition and associated pathological processes has yet to be established. Abnormal SWI signals in the brain of WD patients were previously thought to reflect both iron and copper deposition (Lee et al., 2012; Zhou et al., 2014). But the correlation between copper deposition and CP values should be confirmed by pathological research. Since copper is paramagnetic, it is possible that the observed abnormalities reflect copper deposition (Hingwala et al., 2013). Our data showed that CP values were correlated with both iron and copper content in TX mice. The lack of correlation between pathological parameters and CP values in our series indicates no obvious contribution of myelin or axon degeneration to CP values. SWI can be used to evaluate the deposition of copper and iron in the WD brain, but it cannot reveal pathological changes such as axonal and myelin injury.

We found no correlation between pathological indices such as axonal and myelin injury and metal deposition. This absence of a correlation may suggest that metal deposits are not the only cause of nerve damage; other pathogenic factors may contribute to WD. In our study, we confirmed the presence of abnormal oxidative stress. However, we also found no correlation between these oxidative stress indicators and pathological indicators. Thus, WD nerve injury may be the result of multiple pathogenic factors. In the clinical treatment of WD, we are faced with the dilemma of having a single treatment—copper drainage—for different disease stages. Our study confirmed that there are different pathogenic factors and pathological injury characteristics in different stages of WD disease. This suggests that different treatment schemes, such as copper discharge, reducing oxidative stress, and protecting nerves, should be adopted in WD clinical treatment depending on the different pathogenic factors in different stages.

## 5 CONCLUSIONS

The brain pathological injury characteristics of TX mice at different stages may be different. At the early stage of the disease, there is copper deposition, but no significant pathological damage. In the middle stage of the disease, both copper and iron levels are increased in various brain nuclei, and obvious demyelination and axonal injury, but no obvious neuronal necrosis, can be found in the brains of TX mice. In the late stage of the disease, clear signs of nerve cell necrosis can be seen in the TX mouse brain. Abnormal oxidative stress exists in TX mice at the late stage of disease and may be another pathogenic factor involved in WD nerve injury. Hypointense signals on SWI result from a combination of copper and iron deposition in TX mice, but do not reflect axonal injury or demyelination.

## CONFLICT OF INTEREST

All authors declare that they have no conflicts of interest.

## ETHICAL APPROVAL

All applicable international, national, and/or institutional guidelines for the care and use of animals were followed.

## Data Availability

The data that support the findings of this study are available from the corresponding author upon reasonable request.
